# Control of Cerebral Blood Flow by Blood Gases

**DOI:** 10.3389/fphys.2021.640075

**Published:** 2021-02-18

**Authors:** James Duffin, David J. Mikulis, Joseph A. Fisher

**Affiliations:** ^1^Department of Anesthesia and Pain Management, University of Toronto, Toronto, ON, Canada; ^2^Department of Physiology, University of Toronto, Toronto, ON, Canada; ^3^Thornhill Research Inc., Toronto, ON, Canada; ^4^University Health Network, Toronto, ON, Canada; ^5^Division of Neuroradiology Imaging, Joint Department of Medical Imaging, University Health Network, Toronto, ON, Canada; ^6^Faculty of Medicine, Institute of Medical Science, University of Toronto, Toronto, ON, Canada

**Keywords:** cerebral blood flow, mathematical model, hypercapnia, hypoxia, anemia, hypocapnia

## Abstract

Cerebrovascular reactivity can be measured as the cerebrovascular flow response to a hypercapnic challenge. The many faceted responses of cerebral blood flow to combinations of blood gas challenges are mediated by its vasculature’s smooth muscle and can be comprehensively described by a simple mathematical model. The model accounts for the blood flow during hypoxia, anemia, hypocapnia, and hypercapnia. The main hypothetical basis of the model is that these various challenges, singly or in combination, act via a common regulatory pathway: the regulation of intracellular hydrogen ion concentration. This regulation is achieved by membrane transport of strongly dissociated ions to control their intracellular concentrations. The model assumes that smooth muscle vasoconstriction and vasodilation and hence cerebral blood flow, are proportional to the intracellular hydrogen ion concentration. Model predictions of the cerebral blood flow responses to hypoxia, anemia, hypocapnia, and hypercapnia match the form of observed responses, providing some confidence that the theories on which the model is based have some merit.

## Introduction

The mathematical model proposed here is concerned with the physiological mechanisms regulating cerebral blood flow (CBF) in response to anemia, hypoxia, and hypercapnia and hypocapnia. Cerebral blood flow increases in anemia (Brown et al., 1985; Borzage et al., 2016; Zheng et al., 2016); as well as during acute alterations in arterial blood gases (Willie et al., 2012) such as hypoxia (Cohen et al., 1967; Mardimae et al., 2012), and hypercapnia (Battisti-Charbonney et al., 2011). The model is tested by comparing its predictions to observations.

Cerebral blood flow is largely controlled by changes in the vascular resistance in parenchymal arterioles. The large pial arteries on the surface of the cortex contain multiple layers of vascular smooth muscle cells (Wei et al., 1980). These pial vessels branch into penetrating arterioles containing a single layer of vascular smooth muscle cells (Nishimura et al., 2007), and enter the cortical parenchyma, where micro vessels covered by pericytes but do not control microregional CBF (Hill et al., 2015). Vascular smooth muscle is the final CBF control effector in: (i) *Autoregulation* which mitigates against variations in brain perfusion pressure (Tan and Taylor, 2014; Tzeng and Ainslie, 2014), and (ii) *Neurovascular Coupling* which increases local blood flow in response to increased neuronal metabolic demand (Attwell et al., 2011, 2016; Phillips et al., 2016; Hosford and Gourine, 2019; Hoiland et al., 2020).

Acute changes in arterial blood gases also affect CBF, independently of these regulatory mechanisms (Willie et al., 2012). Hypoxia (Cohen et al., 1967; Mardimae et al., 2012) and hypercapnia (Battisti-Charbonney et al., 2011; Hoiland et al., 2019), as well as decreases in hemoglobin (anemia) (Borzage et al., 2016; Duffin et al., 2020) increase CBF, while hypocapnia decreases CBF (Battisti-Charbonney et al., 2011; Hoiland et al., 2019). It is the increase in CBF during hypercapnia that is the basis of cerebrovascular reactivity (CVR) testing.

In the presence of hypoxia and anemia, vascular tone decreases to increase CBF and maintain an adequate O_2_ supply (Duffin, 2020). Long term changes in CBF occur in chronic anemia (Brown et al., 1985) including sickle cell anemia (Bush et al., 2016), altitude acclimatization (Wolff, 2000) and chronic hypoxia (Powell and Fu, 2008), and are accompanied by a multitude of adaptive changes orchestrated via the HIF 1 alpha pathway (Poellinger and Johnson, 2004). Over the long term the cerebral vasculature remodels to provide larger diameter vessels and accommodate higher CBF (Hulbert et al., 2017).

Vascular smooth muscle tone depends on intracellular [H^+^]; contracting in alkalosis and relaxing in acidosis (Austin and Wray, 1993; Aalkjaer and Peng, 1997) to consequently alter CBF. Changes in intracellular [H^+^] alter intracellular [Ca^2+^] (Swietach et al., 2013) and consequently smooth muscle tone (Boedtkjer, 2018), with the relaxation produced by hypercapnic acidosis, as in CVR testing, mediated by a reduction of [Ca^2+^] (Peng et al., 1998), although, rapid acute acidification can cause a transient increase in intracellular [Ca^2+^] that leads to contraction (Jensen et al., 1993).

Intracellular [H^+^] is the key intracellular regulated ion (Boedtkjer and Aalkjaer, 2012; Boedtkjer, 2018; Rasmussen and Boedtkjer, 2018); with a typical resting intracellular [H^+^] in vascular smooth muscle cells of about 50–80 nM/L (Boedtkjer et al., 2012). This regulation requires a net acid extrusion to maintain normal intracellular acid–base homeostasis, and it is this regulation that is challenged during CVR hypercapnia, producing a vasodilation and increased CBF (Kontos et al., 1977; Peng et al., 1998).

**FIGURE 1:**
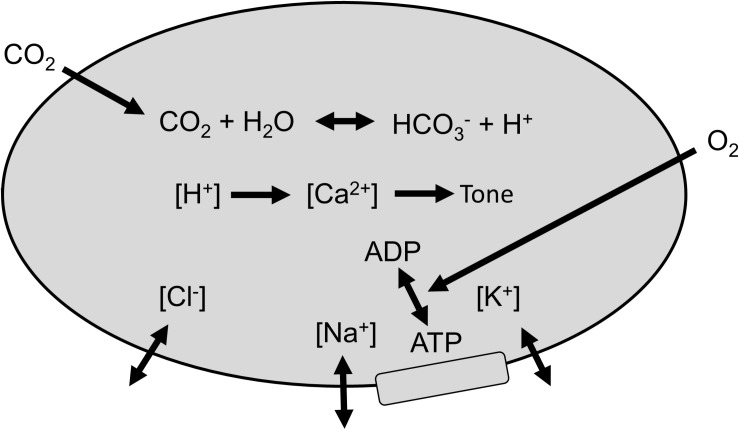
Regulation of smooth muscle [H^+^]. Membrane ion exchangers control strongly dissociated ions, and arterial CO_2_ determines intracellular PCO_2_. Intracellular [H^+^] determines [Ca^2+^] and vascular smooth muscle tone, which controls cerebral blood flow (CBF). O2 is required to fuel the energetic process of controlling [SID].

Stewart (Stewart, 1983; Hughes and Brain, 2013) has suggested an insightful approach to understanding acid-base changes in biological systems. In this system, intracellular [H^+^] is determined by CO_2_ tension, the balance of concentrations of the strongly dissociated ions (the strong ion difference [SID]), and the requirement for electroneutrality. Consequently, the regulation of intracellular [H^+^] depends on cell membrane ion exchangers (Aalkjaer, 1990; Hughes and Brain, 2013; Boedtkjer, 2018; Garneau et al., 2020), to control intracellular [SID]. These membrane ion exchangers are energy dependent (Garneau et al., 2020) so that reductions in O_2_ availability (Hoiland et al., 2016) (blood O_2_ content, CaO_2_) to the cerebral vasculature smooth muscles reduces the ability of these membrane ion exchangers to control intracellular [SID], and hence intracellular [H^+^], smooth muscle tone and CBF. Thus, changes in arterial CO_2_ tension (PaCO_2_) override intracellular [H^+^] regulation to produce vasodilation and vasoconstriction. Similarly, decreases in CaO_2_ caused by decreases in arterial O_2_ tension (PaO_2_, i.e., hypoxia) or hemoglobin concentration ([Hb] i.e., anemia) degrade the efficiency or capacity of membrane ion exchangers that control [SID] producing an increase in intracellular [H^+^], which causes vasodilation and results in an increase in CBF. This model is pictured in Figure 1.

## Materials and Methods

The model equations are presented in the following assumptions using these symbols:

[H^+^] = intracellular hydrogen ion concentration (nM/L).
PCO_2_ = intracellular CO_2_ tension = arterial CO_2_ tension (mmHg).
[SID] = intracellular [strongly dissociated cations] – [strongly dissociated anions] (mM/L).
CBF = cerebral blood flow (ml/min/100 ml).
CaO_2_ = arterial O_2_ concentration/content (ml/ml).

The equation parameters were empirically derived from observed responses to the various disturbances.

Assumption 1: Cerebrovascular smooth muscle intracellular [H^+^] is a function of PCO_2_ and [SID] (Stewart, 1983) (see Appendix):

(1) [H]+(nM/L)=Functionof{PCO,2[SID]}

**FIGURE 2:**
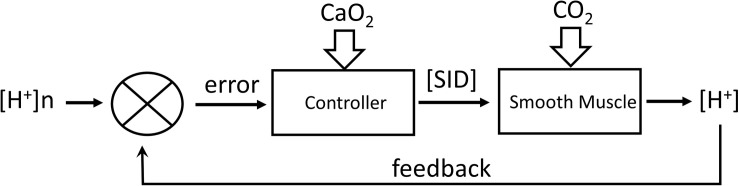
Regulation of [H^+^] by feedback control of [SID] by transmembrane transport of strongly dissociated ions. CO_2_ acts as a disturbance to the system. CaO_2_ acts to alter the controller gain.

Assumption 2: [H^+^] is regulated by a feedback alteration of [SID] proportional to the deviation of [H^+^] from its regulated value of [H^+^]*n* = 40 nM/L according to the following eq. 2, which assumes that maximum vasoconstriction occurs at PCO_2_ = 10 mmHg and [SID] = 5.5 mM/L. Figure 2 outlines the operation of this regulator.

(2) [SID]=5.5+Gain*(([H]+-40)+2)

**FIGURE 3:**
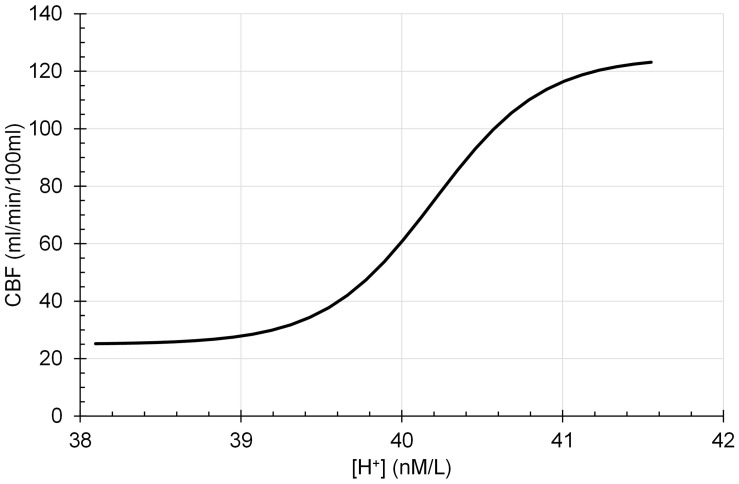
Cerebral blood flow (CBF) is a sigmoidal function of intracellular [H^+^].

Assumption 3: Smooth muscle tone is assumed to be limited to a maximum vasoconstriction and vasodilation so that CBF is a sigmoid function of intracellular [H^+^] according to the following eq. 3, which is illustrated in Figure 3:

(3) CBF=25+100/(1+exp(-([H]+-40.2)/0.34))

**FIGURE 4:**
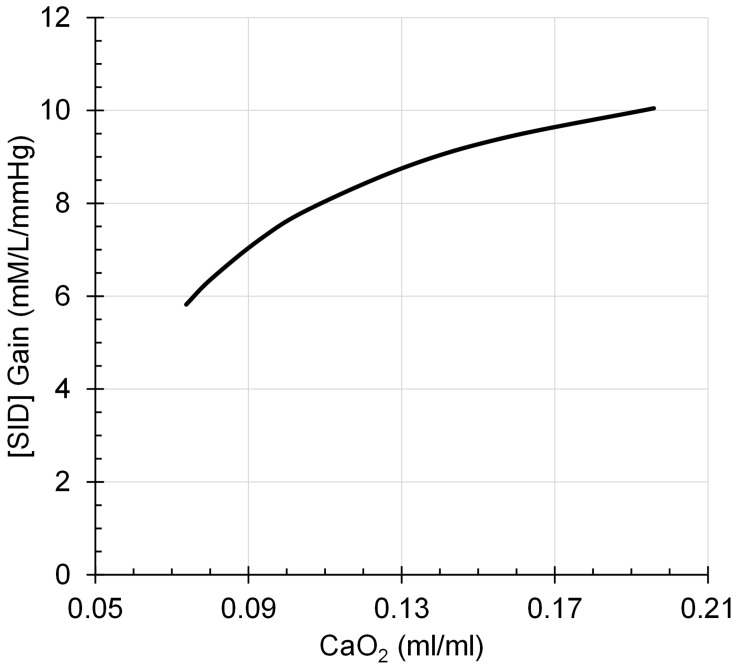
[SID] Gain decreases to a progressively greater degree as CaO_2_ declines in a rectangular hyperbolic function.

Assumption 4: The regulation of [H^+^] by [SID] is oxygen dependent with the Gain of eq. 2 a function of arterial oxygen content, CaO_2_, according to eq. 4, as illustrated in Figure 4.

(4) Gain=12.6-0.5/CaO;2

CaO_2_ is calculated from PaCO_2_, PaO_2_, and [Hb] using previously published equations (Duffin, 2005; Balaban et al., 2013).

**FIGURE 5:**
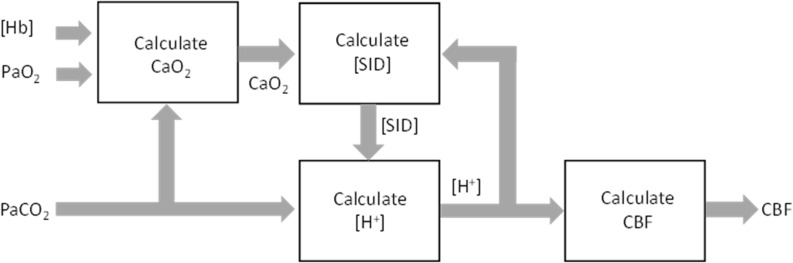
Implementation block diagram of the feedback control of cerebral blood flow (CBF) by PaCO_2_, PaO_2_, and [Hb].

The system equations are solved using an iterative approach (LabVIEW, National Instruments, Austin, TX, United States) outlined in the block diagram of Figure 5.

## Results

**FIGURE 6:**
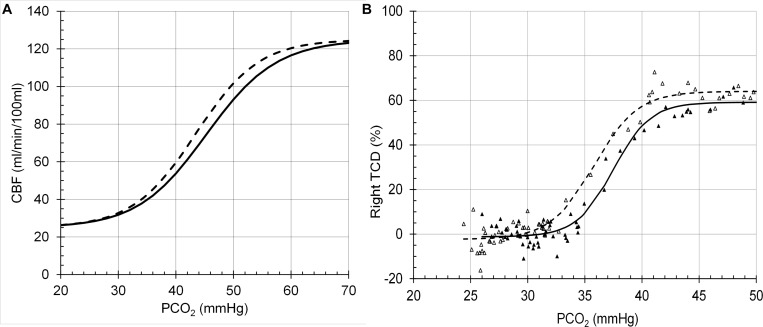
Cerebral blood flow (CBF) responses to PCO_2_ at PO_2_ = 150 mmHg (solid line) and PO_2_ = 50 (dashed line). **(A)** Model response. **(B)** Example transcranial Doppler (TCD) measurements of middle cerebral artery blood flow velocities (Battisti-Charbonney et al., 2011).

**FIGURE 7:**
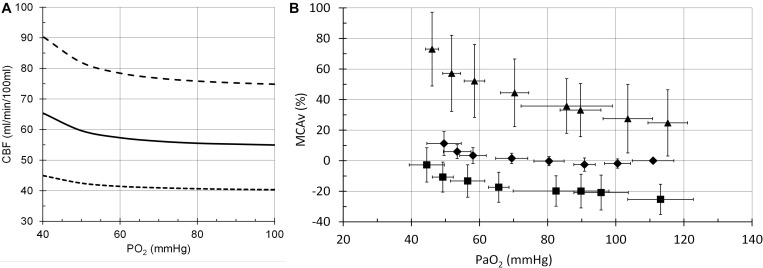
Cerebral blood flow (CBF) responses to PO_2_. **(A)** Model responses at PCO_2_ = 35 mmHg (dotted line), PCO_2_ = 40 mmHg (solid line) and PCO_2_ = 50 mmHg (dashed line). **(B)** Example transcranial Doppler (TCD) measurements of middle cerebral artery blood flow velocities at PCO_2_ = 30 mmHg (squares), PCO_2_ = 40 mmHg (diamonds) and PCO_2_ = 50 mmHg (triangles) (Mardimae et al., 2012).

**FIGURE 8:**
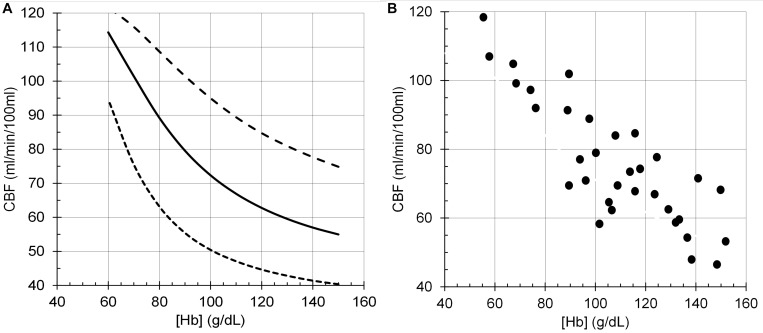
Cerebral blood flow (CBF) responses to anemia ([Hb]). **(A)** Model responses at PCO_2_ = 35 mmHg (dotted line), PCO_2_ = 40 mmHg (solid line) and PCO_2_ = 50 mmHg (dashed line), and PO_2_ = 100 mmHg. **(B)** Data from young end-stage renal disease adults replotted from Figure 1B of Zheng et al. (2016).

The model performance was assessed by its CBF responses to changes in PCO_2_, PO_2_ and [Hb] as shown in the following figures (Figures 6–8), which compare the model responses with examples of observed responses.

## Discussion

The model predictions for CBF responses match the form of the observed responses, providing some confidence that the theories on which the model was based are realistic. Of course, such a model does not prove the theories are correct, merely that they are feasible physiological mechanisms, and the interaction of the flow determinants are generally aligned to that of the model.

It must be emphasised that the model does not attempt to accurately predict the experimental examples provided but is a generic prediction of the form of the responses to test the hypotheses. For example, the midpoint of the sigmoidal response to CO_2_ from a TCD experiment in Figure 6 is lower than that of the model response. However, it is not beyond the bounds of observed midpoints in other experiment using different methods. For example, the midpoints in the TCD experiments (means of 35–36 mmHg) differ from those derived from the vascular resistance which varied over a range of 38–48 mmHg (Duffin et al., 2018). A similar observation may be made for the responses to [Hb] in Figure 8 where the model responses exhibit considerable curvature as [Hb] decreases compared to the experimental observations. We note that there is considerable variability in the experimental observations, and while the authors chose to fit a linear response, the data indicates that curvature is present, as we showed in Duffin et al. (2020). Another confounding factor in the experimental observations is the variability of PCO_2_ in the experiments where PCO_2_ was uncontrolled compared to the model where isocapnia was maintained. In summary, there is no doubt that the model can be improved by adjusting the parameters and the relations used, such as the linear one between [SID] and [H^+^] which is almost certainly a simplification and awaits experimental measurements.

There are several assumptions underlying the model, and, as reviewed in the introduction, some are supported by experimental evidence. That cerebrovascular smooth muscle attempts to maintain a constant intracellular [H^+^] by some regulatory process appears to be supported (Boedtkjer, 2018). Furthermore, that [H^+^] acts via alterations in [Ca^2+^] to control vascular smooth muscle tone and consequently CBF also appears to be substantiated (Aalkjaer and Peng, 1997). The physico-chemical theory of acid-base described by Stewart (Stewart, 1981) and illustrated in Hughes and Brain (2013) dictates that intracellular [H^+^] and bicarbonate [HCO_3_^–^] are dependent variables, with CO_2_ and [SID] independent variables that determine them. Therefore, the model assumption that [H^+^] is regulated by controlling [SID] in the face of a CO_2_ disturbance is an obvious conclusion. That [SID] must be adjusted by membrane transport processes is well known (Garneau et al., 2020).

The simple equation proposed to describe this regulation process is one of many that could be used to describe the relation between [SID] and the error signal, deviation of [H^+^] from a normal resting value. The equation form assumes that maximum vasoconstriction occurs at the hypocapnic limit of vasoconstriction (PCO_2_ = 10 mmHg where [SID] = 5.5 mM/L attempting to restore [H^+^] to 40 nM/L). This assumption has the result that at a normal resting PCO_2_ of 40 mmHg the regulation of [SID] is active and consequently subject to degradation by a lack of O_2_.

The assumption that a decrease of CaO_2_ affects intracellular [H^+^] by degrading the [SID] regulation is a major hypothesis of the model and is testable. That CBF is related to CaO_2_, whether changed by anemia or hypoxia is well documented and the subject of a previous model (Duffin, 2020). Furthermore, it was previously demonstrated that brain tissue PO_2_ was not a viable choice as a sensed variable controlling CBF (Duffin et al., 2020). Consequently, CaO_2_ was the obvious choice of variable to represent a decreased O_2_ supply affecting the supply of energy to the membrane transporters of strongly dissociated ions thereby degrading the [SID] regulation of [H^+^]. However, there does not currently appear to be supportive experimental evidence for this assumption, and it requires investigation.

Other mechanisms by which CaO_2_ may change CBF involving nitric oxide (NO) have been proposed. Some (Hoiland et al., 2020) are linked to neurovascular coupling control of CBF (Attwell et al., 2011) rather than the control of blood O_2_ supply. Others involve release of NO from red blood cells (Hoiland et al., 2016) or endothelial cells (Iadecola, 1992; Kisler et al., 2017). However, experiments show that NO does not appear to be involved in responses to changes in blood gases (White et al., 1998; Ide et al., 2007). Furthermore, these mechanisms for CBF control independent of CO_2_, whether involving NO or not, do not agree with the observations of the interaction of CaO_2_ and CO_2_ in the control of CBF. For example, in hypocapnia, where vasoconstriction lowers CBF, a decrease in CaO_2_ would be expected to increase CBF at least as much as is observed in normocapnia, but does not (Mardimae et al., 2012). The model proposed does so, exhibiting the interaction between CaO_2_ and CO_2_ observed (Figure 5).

## Conclusion

This model can be used to predict the effects of hypoxia, hypercapnia and anemia, alone and in combination, on CBF. It therefore has practical usefulness for intensive care and intra-operative management where these conditions apply, as well as for drugs that affect carbonic acid dissociation and elimination, and shift the oxyhemoglobin dissociation curve.

The hypothetical basis of the model is not only supported in part by the experimental evidence used in its formulation, but also by the success of the model predictions of experimental observations. Thus, the hypothesized mechanisms merit consideration as realistic possibilities, deserving of experimental investigation to determine their correctness. We suggest that the physiological mechanism described in this model for the response to CO_2_ has widespread application as a general principle. It has already been used to describe the responses of the carotid body glomus cells to CO_2_ (Duffin, 2020) and may also be applicable to the central chemoreception of CO_2_ (Guyenet et al., 2019). It is an aspect of a physioc-chemical view of acid-base control pioneered by Stewart (1981) that should enable a better understanding of the processes involved in sensing CO_2_ and the effects of disturbances in energy supply and external CO_2_ on the process. We believe that such an increased understanding will impact research in this field to further characterise the mechanisms involved which in turn may inspire intervention opportunities that apply to clinical care.

## Data Availability Statement

The raw data supporting the conclusions of this article will be made available by the authors, without undue reservation.

## Ethics Statement

Ethical review and approval was not required for the study on human participants in accordance with the Local Legislation and Institutional Requirements. The patients/participants provided their written informed consent to participate in this study.

## Author Contributions

JD: conception and programming. JD, JF, and DM: drafting and revising of the article and final approval of the manuscript. All authors contributed to the article and approved the submitted version.

## Conflict of Interest

JF and DM have equity in Thornhill Medical Inc. JD receives salary support from Thornhill Medical Inc. Thornhill Medical Inc. provided no other support for the study. The authors declare that the research was conducted in the absence of any commercial or financial relationships that could be construed as a potential conflict of interest.

## Appendix

The acid-base control of intracellular [H^+^] was calculated using an iterative approach that solved eq. 1–4 to enforce electrical neutrality by minimizing R.

[HCO]3-=KC*PCO2/[H]+                (1)

[CO]32-=K3*[HCO3]2-/[H]+                (2)

[OH]-=Kw/[H]+                    (3)

R=[SID]+[H]+--[HCO]3--2*[CO]32---[OH]-    (4)

where:

PCO_2_ (mmHg) is an input.
[SID] = [strongly dissociated cations] – [strongly dissociated anions] (mM/L) is an input.
[HCO_3_^–^] (mM/L)
[CO_3_^2–^] (mM/L)
[OH^–^] (nM/L)
KC = 2.45 × 10^–11^
K3 = 1.16 × 10^–10^
Kw = 2.39 × 10^–14^
